# Development and Analysis of Electrochemical Reactor with Vibrating Functional Element for AAO Nanoporous Membranes Fabrication

**DOI:** 10.3390/s22228856

**Published:** 2022-11-16

**Authors:** Urte Cigane, Arvydas Palevicius, Vytautas Jurenas, Kestutis Pilkauskas, Giedrius Janusas

**Affiliations:** 1 Faculty of Mechanical Engineering and Design, Kaunas University of Technology, Studentu Str. 56, 51424 Kaunas, Lithuania; 2 Institute of Mechatronics, Kaunas University of Technology, Studentu Str. 56, 51424 Kaunas, Lithuania

**Keywords:** electrochemical reactor, two-step anodization method, high-frequency excitation method, AAO nanoporous membrane

## Abstract

Nanoporous anodic aluminum oxide (AAO) is needed for a variety of purposes due to its unique properties, including high hardness, thermal stability, large surface area, and light weight. Nevertheless, the use of AAO in different applications is limited because of its brittleness. A new design of an electrochemical reactor with a vibrating element for AAO nanoporous membranes fabrication is proposed. The vibrating element in the form of a piezoceramic ring was installed inside the developed reactor, which allows to create a high-frequency excitation. Furthermore, mixing and vibration simulations in the novel reactor were carried out using ANSYS 17 and COMSOL Multiphysics 5.4 software, respectively. By theoretical calculations, the possibility to excite the vibrations of five resonant modes at different frequencies in the AAO membrane was shown. The theoretical results were experimentally confirmed. Five vibration modes at close to the theoretical frequencies were obtained in the novel reactor. Moreover, nanoporous AAO membranes were synthesized. The novel aluminum anodization technology results in AAO membranes with 82.6 ± 10 nm pore diameters and 43% porosity at 3.1 kHz frequency excitation and AAO membranes with 86.1 ± 10 nm pore diameters and 46% porosity at 4.1 kHz frequency excitation. Furthermore, the chemical composition of the membrane remained unchanged, and the hardness decreased. Nanoporous AAO has become less brittle but hard enough to be used for template synthesis.

## 1. Introduction

The growth rate of the global nanotechnology market is so rapid that it far exceeds the market growth forecasts and is even more impressive because most nanomaterials are still in the early stages of the product life cycle [1]. As well, rapid progress has been made in the field of nanomembrane research, from basic research to the development of next-generation technologies [2]. Ultrathin 2D nanomaterials have many unique physical, electronic, chemical, and optical properties that provide many advantages and distinguish nanomembranes from other nanomaterials [3]. Nanomembranes are a new type of nanomaterial that has great potential and can be applied in a variety of fields, such as biosensors [4,5], chemical sensors [6,7,8], various biomedical applications [9,10], the food industry [11], nano- and microelectromechanical systems [12,13], etc. Nanomembranes can be classified according to the material used in their fabrication [14]. According to the membrane material, synthetic nanomembranes can be organic, inorganic, hybrid, or biological [15]. While developing synthetic nanomembranes, it is important to control their internal structure, because physical and mechanical properties affect the unique properties of the nanomembranes [16]. Different fabrication methods, such as lithography [17], laser machining [18], layer-by-layer deposition [19], sol–gel processing [20], and 3D printing [21,22], are used to create nanoporous structures. These methods require expensive and high-tech equipment; therefore, another well-known alternative method to develop nano-membranes is the electrochemical anodization process [23].

In many applications, control of the geometry of membrane nanopores is a significant criterion [24,25]. Anodic aluminum oxide (AAO) is well known for its large surface area, relatively low cost, well-ordered structure, and ability to control the diameter of nanopores [26]. AAO can be obtained by controlling the parameters of the electrochemical anodization process [27,28]. AAO gradually grows in acidic solutions that anodize aluminum under an electric field between the anode and the cathode. The diameter of AAO pores depends on the anodization potential (voltage), temperature, electrolyte, and concentration [29,30]. The pore diameter and the distance between the pores often increase as the anodization voltage or the electrolyte concentration increases [14,31,32]. Moreover, it has recently been reported that the pore diameter decreases with decreasing electrolyte temperature [27]. According to previous AAO research, the electrochemical anodization process consists of two stages: mild (soft) and hard anodization [26]. In comparison to these two stages, the hard anodization process is more intense, the growth rate of the porous AAO film is higher, and a high amount of heat is released. To obtain nanoscale pores of uniform size, a constant temperature must be ensured throughout the anodization process. Therefore, more requirements for cooling equipment are set.

There are many scientific publications related to the vibrations of nanoporous AAO membrane and their effects in various fields such as microfiltration/nanofiltration [33] or the mitigation of fouling [34]. However, the effect of high-frequency excitation during the anodization process on the geometry of the nanopores in the AAO membrane has not yet been investigated. Vibrations during the electrochemical anodization process can be generated by using a piezoelectric ceramic material, which vibrates during excitation. It is widely used in various applications, such as microfluidic devices [35,36], particle manipulation [37], sensors [38], etc. Lead zirconate titanate (PZT) is an attractive piezoelectric ceramic material with good electromechanical properties; e.g., PZT rings are widely used in the development of various devices [39]. Accordingly, the piezoelectric ceramic ring can be used to control the geometry of nanopores in the membrane during the two-step anodization process. An analysis of the scientific literature [40,41] allows concluding that it is necessary to improve technologies for the development of novel nanoporous AAO membranes.

For most engineering materials, strength and toughness are essential requirements [42]. Ceramics have high hardness and strength due to strong atomic bonds, but strong bonds and the lack of plastic deformation result in ceramic brittleness [43]. To adapt nanoporous AAO to the production of templates [41] or use for ultrasonic nanoimprinting [44], it is important for the oxide to be characterized by nonbrittle nanopore deformation [45]. To create a super thin and more flexible nanoporous membrane from brittle aluminum oxide and to improve the quality of AAO membranes, it is necessary to create a novel electrochemical reactor for the fabrication of AAO membranes by applying high-frequency excitation during the two-step anodization process. 

The aim of this work is to design an electrochemical reactor with a vibrating functional element and to produce nanoporous AAO membranes using the high-frequency ex-citation method.

## 2. Materials and Methods

### 2.1. Design of Electrochemical Reactor

**Figure 1:**
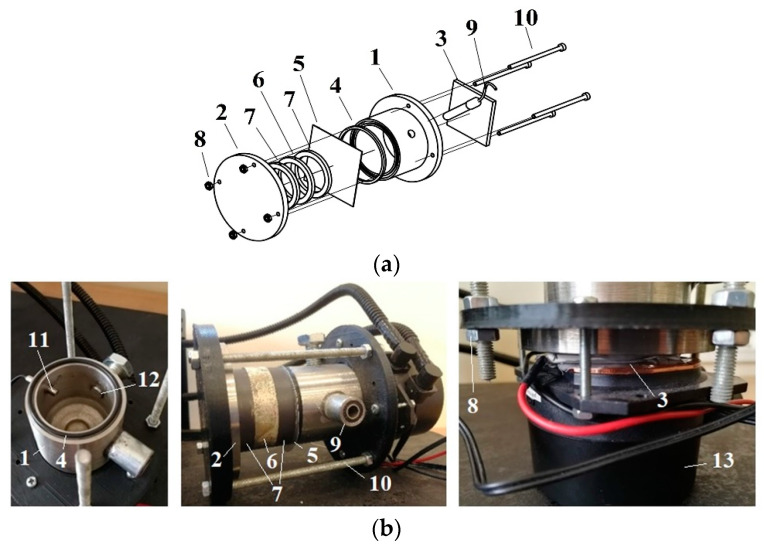
Electrochemical reactor for manufacturing AAO nanoporous membrane. (**a**) Drawing of the electrochemical reactor: 1: corps of the reactor, 2: cover of the reactor, 3: Peltier element, 4: gasket, 5: aluminum sheet, 6: vibrating element (piezoelectric ring), 7: electrical insulating element (piezoceramic), 8: nut M3, 9: mixing device, 10: screw M3x50; (**b**) Construction of the electrochemical reactor: 1: corps of the reactor, 2: cover of the reactor, 3: Peltier element, 4: gasket, 5: aluminum sheet, 6: vibrating element (piezoelectric ring), 7: electrical insulating element (piezoceramic), 8: nut M3, 9: place for mixing device, 10: screw M3x50, 11: temperature sensor, 12: hole for electrolyte filling, 13: cooler.

To design an electrochemical reactor with a vibrating functional element for the fabrication of AAO nanoporous membranes, the effect of high-frequency excitation on the geometry of the nanopores during the anodization process will be investigated. An electrochemical reactor of the novel design is being developed, and its structure is shown in Figure 1.

In terms of reactor structure, all reactor components are assembled through fittings. The reactor corps (Figure 1(1)) and the cover (Figure 1(2)) are composed of AISI 304 stainless steel (this steel does not react with acids during the anodization process). Moreover, as the diameter decreases with decreasing acid temperature, it is important to ensure low temperature during anodization to produce nanoscale pores [46]. For this purpose, the Peltier element (Figure 1(3)) model ‘TEC1 12715’ (12 V; 15 A) is selected. The cooler is connected to the Peltier element and the reactor corps. The Peltier element is lubricated on both sides with a thermal paste, which improves heat exchange and increases the contact area between the cooler, the Peltier element, and the reactor corps. Steel screws (Figure 1(10)) M3 × 50 and steel nuts (Figure 1(8)) are used to reinforce the reactor structure. An aluminum sheet (Figure 1(5)) with a thickness of 0.5 mm is the main material used to manufacture the AAO nanoporous membrane. The aluminum sheet is mounted to the stainless-steel reactor cylinder using an acid-resistant rubber gasket (Figure 1(4)) to prevent liquid leakage. The piezo ring (diameter 50 mm) is clamped on the other side of the aluminum plate. The piezoceramic material PZT 8 is chosen because it is considered to be a good choice for resonant devices [47]. To isolate different anodization and high-frequency excitation currents, the piezoceramic ring (actuator) (Figure 1(6)) is covered on both sides with piezoceramic rings (Figure 1(7)) because the ceramic has impermeability and good transmission of acoustic waves. Finally, the cover and screws are used to assemble all components. It guarantees good contact and vibration transmission during the electrochemical process that will be performed by the two-stage anodization method, which is described in detail in [33]. In addition, a temperature sensor (Figure 1(11)) is installed in the assembled reactor. Because low temperature is a crucial parameter of the anodization process, it is important to ensure an equivalent distribution of electrolytes throughout the reactor volume. Therefore, the mixing impeller is a necessary structural element. The impeller material of the mixing device (Figure 1(9)) is also stainless steel, which is resistant to the electrolyte.

### 2.2. Experimental Setup

**Figure 2:**
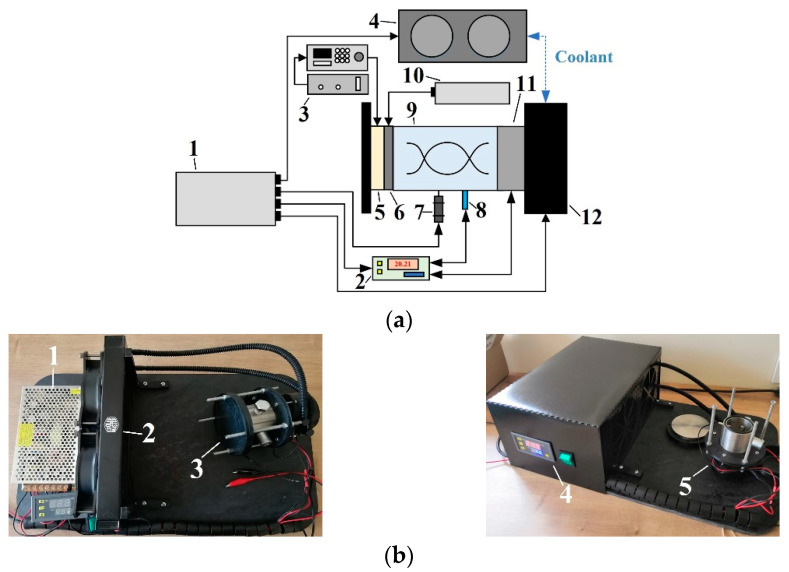
Experimental setup for manufacturing the AAO nanoporous membrane. (**a**) A schematic representation of the experimental setup: 1: DC 1 power unit, 2: temperature control device, 3: voltage amplifier and frequency generator, 4: master cooler radiator, 5: piezoelectric actuator, 6: membrane, 7: mixing device, 8: temperature sensor, 9: electrochemical reactor, 10: DC 2 power unit, 11: Peltier element, 12: master cooler pump; (**b**) Experimental equipment: 1: DC 1 power unit, 2: master cooler, 3: closed electrochemical reactor, 4: temperature control device, 5: open electrochemical reactor.

The experimental setup for manufacturing the AAO nanoporous membrane is presented in Figure 2. Its closed-loop control system consists of the following elements: temperature control device (Figure 2a(2)), temperature sensor (Figure 2a(8)), and Peltier element (Figure 2a(11)). This closed-loop control system is automatic and can be called an automatic control system.

The experimental platform is composed of a 12 V 15 A direct current power supply unit (Figure 2(1)), a temperature control device with a temperature sensor (Digital Thermostat W3001, Juanjuan, China) (Figure 2b(4)), a cooler (Masterliquid lite 240, Cooler Master, Taiwan) (Figure 2b(2)), a 60 V 5 A direct current power supply unit (AN-11808, WEP, Guangdong, China) (Figure 2a(10)), a 12 V mixing device of 108 RPM (JGA25–370 DC Gearmotor, Cnmaway, China) (Figure 2a(7)), a novel electrochemical reactor (Figure 2b(3)), and a 12 V 15 A thermoelectric cooler Peltier element (TEC1 12715, Hebei, China) (Figure 2a(11)).

### 2.3. Experimental Setup for Vibration Measurements

**Figure 3:**
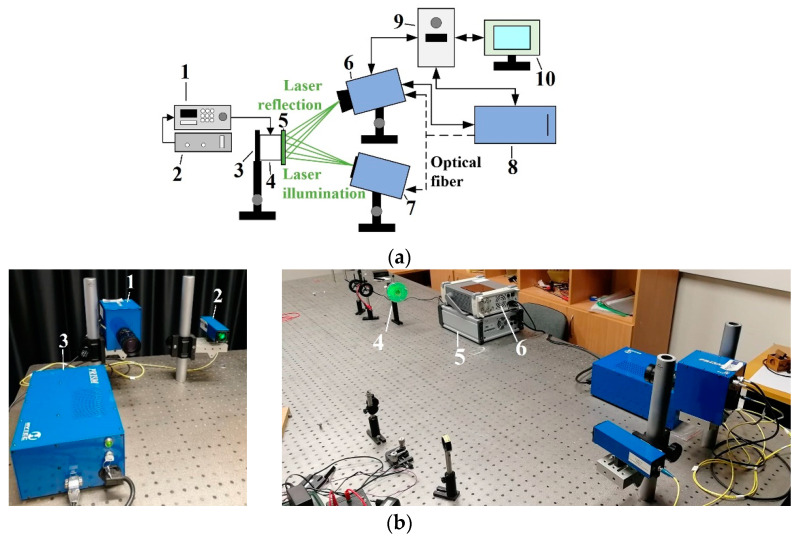
Non-contact holographic measurement system, known as PRISM system. (**a**) Schematic diagram of the PRISM system experimental setup: 1: frequency generator, 2: voltage amplifier, 3: isolated surface for fixing object, 4: piezoelectric actuator, 5: membrane attached to the piezoelectric actuator, 6: camera, 7: illumination head with a green laser, 8: control block, 9: computer, 10: computer screen (image illusion); (**b**) Experimental setup: 1: camera, 2: illumination head with a green laser, 3: control block, 4: membrane attached to a piezoelectric actuator, 5: voltage amplifier, 6: frequency generator.

The method of holography can be used to visualize dynamic processes [33], so the precise real-time instrument for surface measurement—(PRISM) holography system has been used to measure the frequency. The PRISM system (Hytec, Los Alamos, NM, USA) with the piezoelectric actuator is shown in Figure 3. The system consists of deformation equipment (frequency generator and voltage generator), a vibration measurement (holography) unit, and a computer system with software.

A schematic diagram of the experimental setup of the non-contact holographic measurement system is shown in Figure 3a.

Basically, a two-beam speckle pattern interferometer with a green laser has the beam directed at the acting membrane. The object beam is directed at the membrane, and the reference beam is directly captured in the camera. The laser green light (wavelength 532 nm, power 20 mV) is scattered from the object and collected by the camera lens, imaging the object on the camera’s sensors. The image of the object (membrane) is transferred from the camera to the computer. The data on the computer are analyzed by the program PRISM DAQ (Hytec, Los Alamos, NM, USA).

### 2.4. Simulation Method and Conditions of Vibration Process

Vibration simulations of aluminum membrane sheets were performed using COMSOL Multiphysics 5.4 software. The simulation model consisted of the aluminum membrane with a 40 mm diameter and 0.5 mm thickness. The models’ parameters are listed in Table 1.

Moreover, the model was divided into finite tetrahedron elements with fixed support constraint boundary conditions.

### 2.5. Simulation Method and Conditions of Mixing Process

The mixing process simulations were performed using ANSYS 17 software. The model was divided into two zones between which a contact region was created to ensure data integrity. The first zone represented the internal volume of the reactor, and the second zone was for the impeller. The model was meshed by finite tetrahedron elements. The simulation conditions were close to the real ones (water density 998.2 kg/m^3^, viscosity 0.001003 kg/m·s, temperature 20 °C). A four-blade impeller (diameter 20 mm; height 10 mm; blade angle 0 deg.; rotational speed 108 rpm) was used. The k-ε realizable turbulence model was applied to simulate the mixing process. Additionally, gravity was included.

## 3. Results and Discussion

### 3.1. Vibration Analysis

Vibration simulations of aluminum membrane plates were performed using COM-SOL Multiphysics 5.4 software. The vibration modes of the membrane at different frequencies are shown in Table 2.

Table 2 shows that vibrations affect the surface of the membrane sheet because different mode shapes were obtained at different frequencies. In the following descriptions of circular membrane mode shapes, the notation (d, c) means d: the number of nodal diameters, and c: the number of nodal circles. Based on theoretical calculations (Table 2), the first natural frequency mode (0, 1), with a membrane radius of 20 mm (voltage of 200 V), was obtained at the frequency of 3.0 kHz. Displacements on the surface of the membrane sheet were concentrated in the center. As the frequency increased, the second natural frequency mode could be seen. For example, the mode shape (1, 1) was obtained at the frequency of 4.8 kHz. Displacements were visible on both sides of the membrane surface. A membrane surface with four displacement regions was obtained at the frequency of 6.5 kHz.

To confirm the results of the vibration simulation, the described above non-contact holographic measurement system was used (Figure 3). The experimental mode shapes of the membrane at different frequencies are presented in Table 2. The simulation results of the membrane vibrations were verified using the experimental ones. Five similar modes of shapes were obtained by simulation and experiments at the following frequencies: the first natural mode (0, 1) at 3.0 kHz and 3.1 kHz, the second mode (1, 1) at 4.8 kHz and 4.1 kHz, the third mode (2, 1) at 6.5 kHz and 6.3 kHz, the fourth mode (0, 2) at 6.9 kHz and 7.1 kHz, and the fifth mode (3, 1) at 8.8 kHz and 9.1 kHz (Table 2). Comparing the simulation and the experimental results, the errors are estimated due to non-ideal structural stability and material properties and the inaccuracy of the measuring equipment.

### 3.2. Mixing Analysis

**Figure 4:**
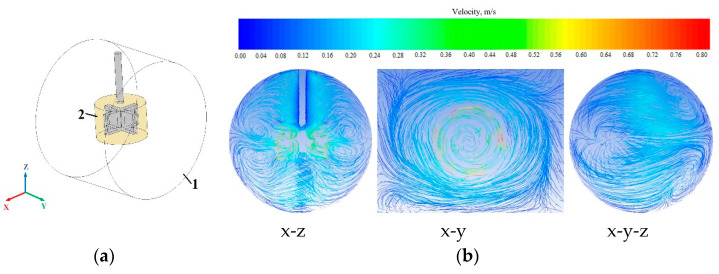

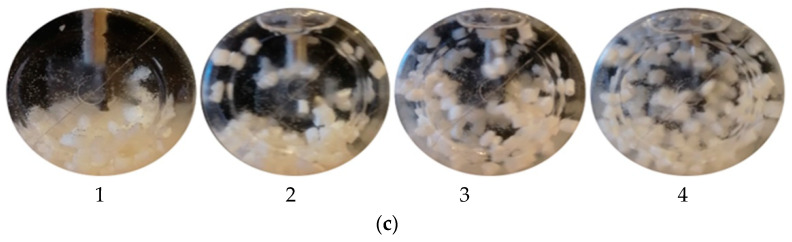
Mixing analysis. (**a**) Simulation model of mixing process (with a coordinate system): 1: internal volume of the reactor (the first zone of the model), 2: impeller zone (the second zone of the model); (**b**) Velocity vectors in different planes; (**c**) Experimental results of mixing process: 1-the mixing process is not running, 2: 0.5 s after the start of mixing, 3: 1 s after the start of mixing, 4: 1.5 s after the start of mixing.

Because low temperature is an important parameter of the anodization process, it is relevant to select a suitable mixing impeller to ensure uniform electrolyte temperature throughout the reactor volume. A mechanical rotary stirrer is often used to create forced flow in the reactor [48]. The mixing process simulations were performed using ANSYS 17 software. The simulation model of the mixing process (with a coordinate system) is presented in Figure 4a. Theoretical velocity vectors in different planes are shown in Figure 4b. The whole volume is mixed. A mixing experiment was performed to confirm the theoretical results. The experimental results of the mixing process are shown in Figure 4c.

Whereas the electrochemical reactor is composed of stainless steel, its corps is not transparent; as a result, the mixing process is not visible. Therefore, it was necessary to create a transparent experimental reactor with a stirred process inside. An experimental model has been developed that corresponds to the dimensions of the real reactor. Water and acrylonitrile butadiene styrene (ABS) thermoplastic polymer pellets that allowed the visualization of particle movement were used in the mixing experiment. ABS polymer was chosen because its density of 1032–1380 kg/m^3^ [49] is similar to that of water. 

For visual analysis, to monitor the distribution of plastic particles throughout the mixing volume, it was decided to use ABS pellets (the size was approximately 2 mm) to occupy approximately one-third of the total reactor volume. Originally, the plastic particles sink into the water. When the mixing process begins, ABS polymer particles begin to move and mix throughout the reactor volume (Figure 4c). The specific motions of the particles were not clearly visible during the experiment, but it is assumed that the mixing experiment is consistent with the theoretical mixing results obtained by the ANSYS 17 software simulation because, in the case of mixing, the particles were distributed and moved throughout the reactor volume.

### 3.3. Temperature Analysis

**Figure 5:**
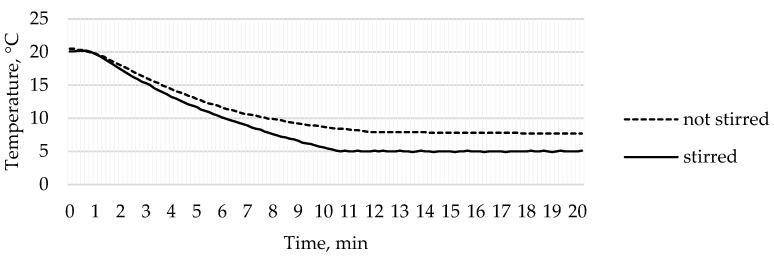
Temperature changes over time inside the reactor.

To create nanoscale pores in the membrane, a low temperature is required inside the electrochemical reactor [46]. For this purpose, an automatic temperature control system was used. During the anodization process, it is crucial to maintain the temperature around 5–8 °C [33]. During the experiment, the temperature of 5 °C in the temperature control device was set. Temperature analysis was performed using two methods: when the liquid inside the reactor was not stirred and when it was stirred. The results of the temperature measurements are shown in Figure 5.

In the case when the liquid in the reactor was not stirred, the uniform temperature of 5 °C was not reached during the experiment. The temperature sensor near the aluminum plate recorded the lowest temperature at 7.7 °C, but as it was set at 5 °C, the automatic control system was constantly working trying to reach the intended temperature, but due to the unmixed liquid, the areas of different temperatures were formed. Additionally, in some places, the temperature in the reactor was significantly lower, and ice formed in the refrigeration zone. Whereas the temperature near the anodization zone was important, the automatic temperature control system could not reach the target temperature of 5 °C when the liquid was not stirred because the temperature distribution in the reactor’s volume was insufficient for the electrochemical process. When the liquid in the reactor was stirred, the temperature inside the reactor ranged from 4.5 °C to 6.0 °C with a functioning automatic temperature control system. Such temperature changes are permissible during the anodization process. Therefore, the temperature control system is suitable for the design of the reactor as it provides the ability to maintain the required temperature during electrochemical processes. The results of the temperature analysis confirm that the mixing process is important in maintaining a constant liquid temperature throughout the reactor volume.

### 3.4. Fabrication of AAO Nanoporous Membrane

A high-purity aluminum alloy (1050A, 99.5%) sheet of 0.5 mm thickness was the main material used for the fabrication of the AAO nanoporous membrane (radius 20 mm). For fabrication, the experimental setup for the two-step anodization process (Figure 2) was used. 

For the experiment, the aluminum sheet was cut into square specimens (5 cm × 5 cm). The specimens were annealed at 400 °C for 4 h in a conventional furnace in a nitrogen atmosphere. After that, the specimens were degreased in acetone. In the novel electrochemical reactor with a vibrating functional element, the aluminum sheet was used as the anodic electrode, whereas the corps of the reactor was used as the cathodic electrode. In the first-step anodization, the aluminum sheet was anodized at 60 V and 5 °C temperature for 1 h in 0.3 M oxalic acid (H_2_C_2_O_4_) electrolyte. After the first-step anodization, the obtained oxide layer was removed by chemical etching in a mixture of 3.5% concentrated phosphoric acid (H_3_PO_4_) and 2% chromium anhydride (CrO_3_) acid solution (by volume) in water at 20 °C for 1 h. After etching, the specimen was rinsed with distilled water. Then, the second anodization was carried out on the same aluminum sheet at 60 V and 5 °C temperature for 8 h in 0.3 M oxalic acid. Then, the specimen was removed from the reactor, rinsed with distilled water, and dried in air.

The same procedure was performed in the two-step anodization process using high-frequency excitation. The first and second mode shape methods were chosen to monitor for significant changes in the AAO nanoporous membranes. The frequency was set at 3.1 kHz and 4.1 kHz, respectively.

**Figure 6:**
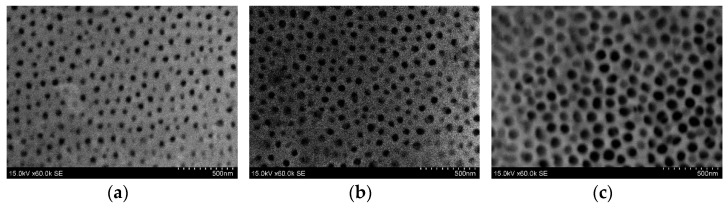
SEM images of AAO nanoporous membrane. (**a**) AAO nanoporous membrane after the two-step anodization process; (**b**) AAO nanoporous membrane after the two-step anodization process using 3.1 kHz frequency excitation; (**c**) AAO nanoporous membrane after the two-step anodization process using 4.1 kHz frequency excitation.

AAO morphology and surface chemical composition were determined by scanning electron microscopy (SEM) and energy dispersive spectroscopy (EDS), respectively. The Hitachi S-3400N scanning electron microscope with an integrated Bruker energy dispersive X-ray spectroscopy (EDS) system was used. SEM images of AAO nanoporous membranes are shown in Figure 6.

Using the image processing program “ImageJ”, the pore diameter (D_p_) and the distance between the pores (D_c_) were determined. To characterize the AAO structure of nanopores, the parameter of porosity was used. Porosity (P) can be defined as the ratio of the surface area occupied by pores and the whole surface area. The P value can be calculated as follows [50]:

P = 0.907 · (D_p_/D_c_)^2^%, (1)

where D_p_: pore diameter, D_c_: interpore distance.

The morphological parameters (D_p_, D_c_, and P) of the nanoporous membranes are shown in Table 3.

The obtained AAO membrane had a pore diameter of 55.0 ± 10 nm, the interpore distance of 121.4 ± 20 nm, and 19% porosity. Using the frequency excitation of 3.1 kHz, the obtained AAO membrane had a pore diameter of 82.6 ± 10 nm, an interpore distance of 120.0 ± 20 nm, and 43% porosity. Likewise, using frequency excitation of 4.1 kHz, the obtained AAO membrane had a pore diameter of 86.1 ± 10 nm, an interpore distance of 120.5 ± 20 nm, and 46% porosity. Overall, these results reveal that, when frequency excitation is used, the pore diameter increases, leading to an increase in porosity. However, the interpore distance has been found to be independent of the frequency.

Analysis of energy dispersive X-ray spectroscopy (EDS) allowed the qualitative and quantitative determination of chemical composition. The chemical compositions of nanoporous AAO are shown in Table 4. The analysis showed that Al_2_O_3_ predominates. Other peaks showed a lower carbon and sulfur content (carbon and sulfur are impurities due to amorphous anodic aluminum oxide). There are no significant changes in the chemical composition of AAO membranes. Thus, the elemental composition of porous membranes has been found to be independent of the frequency. Moreover, the detailed chemical composition indicates the successful fabrication of AAO using frequency excitation.

Since the porosity of the oxide was increased by frequency excitation, it is important to perform hardness measurements to confirm that the porosity affects the mechanical properties of aluminum oxide.

The hardness measurements were taken with Vickers indentations with a diamond tip (Micro Vickers Hardness Testing Machine: HM-200, Mitutoyo, Japan). Each specimen was measured at least five times, and the average was taken. The hardness measurements were recorded in software and represented in Vickers hardness units. 

The hardness tests showed a decrease in hardness from 4.73 GPa to 1.40 GPa comparing the AAO nanoporous membrane after the two-step anodization process without frequency excitation and AAO nanoporous membrane after the two-step anodization process with frequency excitation at 4.1 kHz. Studies performed by other researchers have shown similar results in which the hardness value depends on porosity. The hardness shows a decreasing trend with increasing porosity [51]. In another study, hardness measurements revealed no significant cracks around the indentation [52]. Furthermore, only minor cracks between the pores could be observed inside the indentation [53]. 

Thus, the obtained results reveal that under the same anodization conditions and using high-frequency excitation, the hardness of the AAO membrane decreases, and the porosity increases. Analyzing the brittleness of the AAO membrane, no additional studies were performed, but based on the researchers’ insights that as porosity increases and hardness decreases, brittleness also decreases, we assumed that the AAO membrane produced using high-frequency excitation was less brittle, but hard enough to be used for template synthesis or other applications. 

Furthermore, considering the theoretical model of the porous aluminum growth mechanism, it can be assumed that the resonant frequency excitation to the AAO nanoporous membrane promotes better mixing of the electrolyte on the oxide and electrolyte interface. At the oxide/electrolyte interface, the electrolyte concentration is constantly renewed. As a result, high-efficiency oxide growth is obtained. In the future, theoretical calculations should be performed to confirm the theory of high-frequency excitation during the anodization process and the effect of high frequency on the growth mechanism of porous aluminum oxide. 

## 4. Conclusions

The development and analysis of an innovative electrochemical reactor with a vibrating element are presented. The reactor to produce nanoporous AAO membranes by the two-step anodization method was proposed. To produce less brittle oxide, the high-frequency excitation method was used; therefore, a vibrating element (piezoceramic ring) was integrated into the reactor’s structure. It generates vibrations in the aluminum sheet during anodization. Whereas it is necessary to ensure a temperature of 5–8 °C during the electrochemical process, a Peltier element and a temperature control system were installed in the reactor to ensure the uniform temperature of the liquid throughout the reactor’s volume. The reactor also includes a mixing system with a four-blade impeller. In addition, the reactor’s corps was composed of stainless steel to ensure its resistance to electrolytes.

Vibration, mixing, and temperature analyses were performed. Mathematical models were simulated using COMSOL Multiphysics 5.4 and ANSYS 17 software. Theoretical calculations were experimentally verified. The following results of the reactor design analysis were obtained:

- The high-frequency excitation method was used during the vibration experiment. Five vibration mode shapes were obtained at different frequencies: the first mode shape (0, 1) at 3.0 kHz and 3.1 kHz, the second mode shape (1, 1) at 4.8 kHz and 4.1 kHz, the third mode shape (2, 1) at 6.5 kHz and 6.3 kHz, the fourth mode shape (0, 2) at 6.9 kHz and 7.1 kHz, and the fifth mode shape (3, 1) at 8.8 kHz and 9.1 kHz. The simulation and the experimental results of membrane surface displacements were close, but not identical, because of nonideal structural stability and material properties and the inaccuracy of the measuring equipment;
- It was found that the designed impeller was sufficient for the mixing process. The whole volume in the reactor was mixed. However, specific particle motions were not clearly captured during the experiment. When the mixing device was turned off, the particles did not move throughout the reactor volume, but in the case of the mixing process, the particles were distributed throughout the reactor volume. It was assumed that the mixing experiment was related to the simulation results;
- In the case where the liquid in the reactor was not stirred, the uniform temperature of 5 °C was not reached during the experiment. In addition, in some places, the temperature in the reactor was significantly lower, and cold zones with ice were formed. The temperature sensor recorded the lowest temperature of 7.7 °C. In the case where the liquid was stirred inside the reactor, the temperature ranged from 4.5 °C to 6.0 °C using an automatic temperature control system. Such temperature changes were acceptable.

The results of the vibration, mixing, and temperature analysis confirmed that the design of the novel electrochemical reactor met the requirements. Analyses have shown that the use of the high-frequency excitation method offers a real opportunity to develop functional nanoporous AAO membranes. A novel aluminum anodization technology, which uses high-frequency excitation in the two-step anodization process, results in AAO membranes with 82.6 ± 10 nm pore diameters and 43% porosity using frequency excitation at 3.1 kHz and AAO membranes with 86.1 ±10 nm pore diameters and 46% porosity using frequency excitation at 4.1 kHz. The chemical composition of the membranes remained unchanged, but the pore diameter increased, resulting in higher porosity and lower hardness. It can be assumed that the nanoporous AAO has become less brittle but hard enough to be used for template synthesis. 

The results obtained under the controlled and well-described two-step anodization process with high-frequency excitation conditions will be useful in synthesizing and improving the structure and quality of AAO nanoporous membranes.

## Figures and Tables

**Table 1:** Models’ parameters of vibration process.

Parameter	Unit	Values
Membrane radius	mm	20
Membrane thickness	mm	0.5
Material Young’s modulus	MPa	68,000
Material mass density	kg/m^3^	2712
Material Poisson’s ratio	-	0.33
Radial direction pretension load	MPa	38
Common factor in natural frequency	Hz	1256
1st natural frequency mode, mode shape (0, 1)	Hz	3020
2nd natural frequency mode, mode shape (1, 1)	Hz	4812
3rd natural frequency mode, mode shape (2, 1)	Hz	6450
4th natural frequency mode, mode shape (0, 2)	Hz	6933
5th natural frequency mode, mode shape (3, 1)	Hz	8835

**Table 2:** Surface displacement field of the membrane at different frequencies (at 200 V, with radius 0.020 m).

**Simulation Results by Using COMSOL Multiphysics 5.4 Software**				
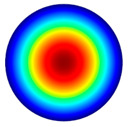 3.0 kHz	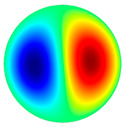 4.8 kHz	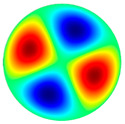 6.5 kHz	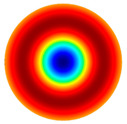 6.9 kHz	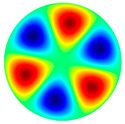 8.8 kHz
**Experimental Results by Using the PRISM System**				
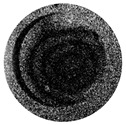 3.1 kHz	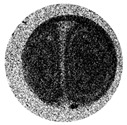 4.1 kHz	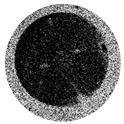 6.3 kHz	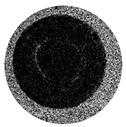 7.1 kHz	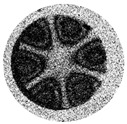 9.1 kHz

**Table 3:** Morphological parameters (D_p_, D_c_, and P) of the AAO nanoporous membrane obtained during the two-step anodization process.

Parameter	No Frequency Excitation	Frequency Excitation at 3.1 kHz	Frequency Excitation at 4.1 kHz
Pore diameter (nm)	55.0 ± 10	82.6 ± 10	86.1 ± 10
Interpore distance (nm)	121.4 ± 20	120.0 ± 20	120.5 ± 20
Porosity (%)	19	43	46

**Table 4:** Chemical composition of AAO nanoporous membrane.

Element	No Frequency		Frequency Excitation at 3.1 kHz		Frequency Excitation at 4.1 kHz	
	Atomic Concentration, at%	Error, %	Atomic Concentration, at%	Error, %	Atomic Concentration, at%	Error, %
Carbon	1.37	0.2	2.02	0.5	1.36	0.3
Oxygen	62.77	6.1	65.18	7.8	62.69	6.6
Aluminum	35.57	2.4	32.51	2.4	35.67	2.3
Sulfur	0.30	0.0	0.29	0.1	0.29	0.1

## Data Availability

Not applicable.
